# Implementation and Evaluation of a Home-Based Pre-Exposure Prophylaxis Monitoring Option: Protocol for a Randomized Controlled Trial

**DOI:** 10.2196/56587

**Published:** 2024-09-23

**Authors:** Chase Cannon, Katherine Holzhauer, Matthew Golden

**Affiliations:** 1 Department of Medicine Division of Allergy & Infectious Disease University of Washington Seattle, WA United States; 2 HIV/STI/HCV Program Public Health – Seattle & King County Seattle, WA United States; 3 Sexual Health Clinic Harborview Medical Center University of Washington Seattle, WA United States

**Keywords:** pre-exposure prophylaxis, self-collection, telehealth, sexually transmitted infections, implementation, evaluation, HIV prevention, HIV, PrEP, home-based pre-exposure prophylaxis, HB-PrEP, stigma, home option testing, sexual health, impact, gender, sexual minorities, race, human immunodeficiency virus, United States, sexual minority men, SMM, barriers, barrier, clinical monitoring, monitoring

## Abstract

**Background:**

HIV prevention is a public health priority. Despite progress in recent years, pre-exposure prophylaxis (PrEP) use remains suboptimal especially among groups disproportionately impacted by new HIV diagnoses such as gender and sexual minorities of color. Multiple barriers including a lack of PrEP providers and challenges with attending quarterly monitoring visits contribute to low PrEP uptake and retention. Home-based PrEP (HB-PrEP) services could reduce stigma, increase convenience, expand health system capacity for PrEP care, and improve PrEP retention.

**Objective:**

Home Option Testing for PrEP (HOT4PrEP) is a hybrid randomized controlled trial (RCT) that aims to examine whether HB-PrEP care is acceptable to PrEP users, feasible to implement in a sexual health clinic setting, and impacts PrEP retention.

**Methods:**

The RCT will recruit 458 persons currently taking or soon to initiate PrEP at a sexual health clinic in Seattle, Washington, and randomize them to continue the standard of care or have the option to use HB-PrEP for 2 of 3 triannual PrEP follow-up visits. Participants in the intervention arm will be sent home kits containing gonorrhea and chlamydia swabs and Tasso devices for blood self-collection. The primary outcome is PrEP retention between groups at 20 months; secondary outcomes include user satisfaction and acceptability, feasibility, self-reported PrEP adherence, and sexually transmitted infection (STI) incidence. Interviews with PrEP users and clinic staff will elucidate barriers and facilitators of implementation.

**Results:**

The HOT4PrEP RCT began enrolling in March 2022, was on hold during the height of the US mpox epidemic, then resumed enrollment in December 2022. Of the first 100 enrollees, the median age is 34 years, and most are cisgender gay men (89/100, 89%) with at least some college education (91/100, 91%). Among the 49 participants randomized to the HB-PrEP option, 33 (67%) chose to self-collect samples at home at least once, of whom 27 (82%) successfully returned test kits for HIV and STI testing. Primary PrEP retention and qualitative analyses are ongoing.

**Conclusions:**

Implementation of HB-PrEP into a high-volume sexual health clinic seems to be feasible and acceptable to early RCT enrollees. This strategy has the potential to address individual and systemic barriers associated with initiating and persisting on PrEP, such as increasing sexual health agency and expanding clinical capacity to serve greater numbers of PrEP users.

**Trial Registration:**

ClinicalTrials.gov NCT05856942; https://clinicaltrials.gov/study/NCT05856942

**International Registered Report Identifier (IRRID):**

DERR1-10.2196/56587

## Introduction

### Background

In 2021, the majority of new HIV infections in the United States occurred in sexual minority men [1]. The US federal government targeted resources toward the goal of reducing new HIV infections by 75% by the year 2025 through the “Ending the HIV Epidemic” initiative [2] and highlights the prevention of new infections as a key component of the plan. While pre-exposure prophylaxis (PrEP) has been proven to reduce new HIV diagnoses at the population level [3,4], it remains an underused intervention. Preliminary evidence suggests that only 36% of people in the United States with an indication for PrEP received a prescription in 2022, up only slightly from 30% in 2021 [5]. Though PrEP coverage among sexual minority men at greatest risk for HIV in Seattle-King County, Washington approached 65% in 2021 [6], PrEP use remains low among younger people and some sexual minority men of color [4,6]. As part of its Ending the HIV Epidemic initiative, Public Health-Seattle and King County (PHSKC) aims to achieve ≥70% PrEP coverage among sexual minority men and transgender persons, with equal use among all racial and ethnic groups.

Structural barriers such as lack of access to PrEP medical providers and the burden of quarterly clinical monitoring visits have stymied PrEP scale-up in the United States for years [7-9]. PrEP retention (defined as maintaining all aspects of PrEP care, including attending follow-up visits and obtaining monitoring tests every 3 months according to US guidelines [10]) represents a substantial gap in the PrEP care continuum [11,12]. Studies on national PrEP retention rates suggest only 56% of individuals who initiate PrEP remain on PrEP for at least 1 year, and only 41% persist through year 2 [13]. Younger age, Black race, and unstable or lower income are each associated with discontinuations and being lost to follow-up [14]. We have observed similar levels of PrEP retention in the PHSKC Sexual Health Clinic (SHC), with 40% of PrEP users discontinuing the intervention at least once within 12 months [15]. The COVID-19 pandemic created dramatic reductions in sexual health service capacity [16] that only further exacerbated and continues to impact existing structural barriers that lead to lower PrEP access and higher numbers of discontinuations [17-19]. Among sexual minority men newly diagnosed with HIV in King County, previous PrEP use and discontinuations are common and underscore the urgent need to develop and implement novel targeted strategies to minimize barriers and provide individuals with more options to stay on PrEP [20].

Home-based PrEP (HB-PrEP), whereby an individual self-collects PrEP monitoring specimens at home, mails them into a laboratory for testing, and follows up with a provider remotely to renew PrEP prescriptions, has the potential to decrease PrEP access barriers and increase PrEP continuation rates. Studies confirm that patients are comfortable self-collecting bacterial sexually transmitted infection (STI) screening specimens [21] and that HB-PrEP monitoring is acceptable and often preferable to in-person care [11,22,23]. Configuring PrEP programs to users’ needs can optimize both uptake and delivery [24]. Particularly for young sexual minority men, programs that are convenient, discreet, and provide them with agency over PrEP use and monitoring may reduce the stigma associated with PrEP use, and increase PrEP uptake and retention [11,23]. Furthermore, HB-PrEP may decongest high-volume clinical sites and create additional capacity to grow PrEP programs.

Several direct-to-consumer companies [25] and fewer research or clinic-based PrEP programs provide PrEP services completely from home [22,23,26]. However, opportunities to adequately screen for newly acquired syphilis and HIV in the home setting are often limited. Given that STI incidence is high among people on PrEP, [27] the US Centers for Disease Control and Prevention guidelines recommend PrEP users be screened for STI quarterly, specifically for syphilis and HIV using assays with narrow windows from infection to test positivity [12]. Syphilis is highly associated with HIV acquisition in PrEP users; [28] thus, it is important that PrEP monitoring include tests that can distinguish new from old syphilis, such as with the quantitative rapid plasma reagin (RPR). Approximately 40% of PHSKC PrEP users have a history of syphilis, of which 25% are serofast (have a persistently positive RPR despite treatment in the past) and require a quantitative RPR for syphilis screening [29]. Although previous HB-PrEP studies used procedures that allowed PrEP users to collect all specimens (gonorrhea and chlamydia [GC and CT] swabs and blood samples) from home [22,26], nearly all require fingerstick specimens to perform a less accurate rapid HIV antibody test and collected insufficient blood volume to perform RPR titers, limiting the ability to determine whether titers were stable or indicative of newly acquired syphilis. We previously conducted a pilot study [29] that showed the acceptability and feasibility of using a novel blood self-collection device (formerly Tasso OnDemand, second-generation larger volume device Tasso+) [30] to obtain samples suitable for HIV antigen and antibody and quantitative RPR testing. However, whether these devices could be used successfully within a dedicated program for serial PrEP monitoring remains unknown.

### Objectives

Through a hybrid [31] randomized controlled trial (RCT), we aim to simultaneously evaluate the effectiveness of HB-PrEP as a strategy for improving PrEP retention and its implementation into an urban SHC’s existing PrEP program.

The specific aims of our study are to: (1) evaluate the impact of HB-PrEP on PrEP retention rates among groups assigned to either home-based monitoring or routine care and (2) assess the program’s reach and define the factors that influence HB-PrEP implementation.

## Methods

### Study Overview

The HOT4PrEP (Home Option Testing for PrEP) project is a series of interrelated studies designed to implement and evaluate an HB-PrEP monitoring system that includes the option for biological specimen self-collection at home and remote clinical follow-up in Seattle-King County, Washington. A detailed description of Tasso device use and results of preimplementation acceptability and feasibility analyses are published elsewhere [29]. The current phase of the HOT4PrEP project is an explanatory sequential mixed methods study consisting of 2 components, that are (1) a hybrid RCT wherein we evaluate HB-PrEP as an implementation strategy and its effect on PrEP retention over time along with other secondary outcomes, and (2) semistructured interviews with study participants and clinic staff to explore individual and system-level barriers and facilitators to HB-PrEP implementation.

For the first study component, we will enroll 458 participants from the PHSKC SHC and randomly assign participants 1:1 to either continue the PrEP standard of care ([SOC], control group) or to have an HB-PrEP monitoring option (intervention). Participants in both arms will complete serial web-based surveys and receive PrEP follow-up through telehealth or at the clinic (Textbox 1). At the time of trial conception, monitoring intervals were initially set at every 3 months; however, these changed to every 4 months in March 2023 following the SHC PrEP program’s shift to reduce the total number of required annual visits. HB-PrEP participants receive mailed sampling kits containing instructions and materials to collect extragenital (pharyngeal and rectal) GC and CT swabs and 2 devices to self-collect capillary blood from the upper arm into microtainer tubes. Participants mail kits through United Parcel Service to the PHSKC laboratory for creatinine, quantitative syphilis serologies, and fourth-generation HIV antigen/antibody testing. Secondary outcomes include reach (number of kits requested and used per participant), user satisfaction with the assigned PrEP monitoring program, STI incidence, and time from resulting of abnormal laboratory values to participant notification. Participation in this first RCT component is not incentivized.

For the second component of the study, we will use quantitative data from surveys to inform semistructured interview guides. In-depth interviews will take place with study participants, staff, and administrative personnel to determine factors associated with the program’s implementation.

**Study procedures for the home option testing for pre-exposure prophylaxis randomized controlled trial.**

The home option testing for pre-exposure prophylaxis trial is a hybrid implementation study that randomizes new or current HIV pre-exposure prophylaxis (PrEP) patients at the Sexual Health Clinic in Seattle, Washington, to have the option of home-based monitoring with self-collection of samples for HIV, syphilis, gonorrhea, chlamydia, and creatinine and telehealth follow-up vs standard care including in-clinic visits and monitoring. Trial enrollment began in March 2022 and the primary outcome is pre-exposure prophylaxis retention at 20 months.

**Baseline visit (in-person visit required)**

- Intervention arm: Home-based pre-exposure prophylaxis (HB-PrEP) participants
  Complete informed consent and enrollment.
  Confirm baseline laboratory tests are complete (hepatitis A total b, hepatitis B sAb and sAg).
  Ensure interval monitoring laboratory tests are collected (HIV Ag and Ab enzyme immunoassay (EIA), serum creatinine, quantitative vs quantitative rapid plasma reagin (RPR) based on history of previous syphilis).
  Participant completes web-based survey.
  Provide 30-day PrEP supply with 3 refills.
  Perform home kit teaching.
- Standard of care (SOC) arm: Routine care participants
  Complete informed consent, enrollment.
  Confirm baseline laboratory tests are complete (hepatitis A total b, hepatitis B sAb and sAg).
  Ensure interval monitoring laboratory tests are collected (HIV Ag and Ab EIA, serum creatinine, quantitative versus quantitative RPR based on history of previous syphilis).
  Participant completes web-based survey.
  Provide 30-day PrEP supply with 3 refills.

**2-4 days later**

- Review laboratory results: if HIV or HBsAg is positive, bring the participant to the sexual health clinic for clinical evaluation.

**Month 4, 8, and 16 visits**

- Intervention arm: HB-PrEP participants (Follow-up by telehealth)
  Study staff (PrEP disease research intervention specialist or other trained staff)
    Assess adherence.
    Confirm no symptoms of acute HIV or contact with sexually transmitted infection (STI).
    Routine PrEP counseling.
    Order applicable PrEP laboratory tests (HIV Ag and Ab EIA, RPR qual or quant, gonorrhea and chlamydia [GC and CT], creatinine).
    Troubleshoot home kit issues.
    Reminder to return kit with self-collected samples.
    Reminder to complete web-based survey.
    Provide 30-day PrEP supply with 3 refills.
    Schedule or give reminder for next visit.
- SOC arm: Routine care participants
  Study staff (PrEP disease and intervention specialists [DRISs] or other trained staff)
    Confirm no signs or symptoms of acute HIV and STI or known sexual contact with a person diagnosed with an STI.
    If STI contact or concern for acute HIV and STI: notify the clinician, principal investigator (PI), and research coordinator and arrange an in-person evaluation.
    Draw blood; participant self-collects GC and CT specimens at exposed sites (pharyngeal, vaginal, and rectal).
    Order and process specimens for HIV Ag and Ab EIA, GC/CT, RPR qual or quant, creatinine (month 6 only). Provide 30-day PrEP supply with 3 refills.
    Participant completes web-based survey.
    Schedule or give reminder for next visit.

**Month 12 visit (in-person visit required)**

- PrEP DRIS
  Confirm no signs or symptoms of acute HIV and STI or known sexual contact to a person diagnosed with an STI.
  If STI contact or concern for acute HIV and STI - notify clinician, PI and research coordinator and arrange in-person evaluation.
  Draw blood; participant self-collects GC and CT specimens at exposed sites (pharyngeal, vaginal and rectal).
  Order & process specimens for HIV Ag and Ab EIA, GC and CT, RPR qual or quant, creatinine (may defer if already done within last 3 months).
  Provide 30-day PrEP supply with 3 refills.
  Participant completes web-based survey.
  Schedule or give reminder for next visit.
  For HB-PrEP or intervention arm enrollees: Troubleshoot home kit issues, provide kit for next visit if desired.
- Clinician
  Assess adherence, side effects, willingness to continue PrEP.
  Address any participant concerns about PrEP. If issues are related to the study, refer to the research coordinator or PI directly.
  Complete hepatitis A or B or other vaccines (eg, HPV) if indicated.
  Provide 30-day PrEP supply with 3 refills.

**Month 20 study exit visit (in-person visit required)**

- PrEP DRIS
  Confirm no signs or symptoms of acute HIV and STI or known sexual contact with a person diagnosed with an STI.
  If STI contact or concern for acute HIV or STI: notify the clinician, PI, and research coordinator and arrange an in-person evaluation.
  Draw blood; participant self-collects GC and CT specimens at exposed sites (pharyngeal, vaginal, and rectal).
  Order and process specimens for HIV Ag and Ab EIA, GC and CT, RPR qual or quant, creatinine (may defer if already done within previous 4 months).
  Provide 30-day PrEP supply with 3 refills.
- Exit assessment: participant will either complete web-based exit survey or may be selected to participate in exit interview with the research coordinator or PI.

### Study Setting and Staff

The PHSKC SHC provides direct medical services related to sexual health and STI to over 6500 patients each year and is the largest single diagnosing site for HIV infection in the Pacific Northwest region of the US. Of all patients seen annually in the SHC, approximately 41% identify as sexual minority men and 42% as racial or ethnic minorities. Disease and research intervention specialists (DRIS), who are nonclinical public health staff trained in HIV and STI prevention and to provide partner services, currently serve as PrEP navigators and conduct all PrEP initiation and nonclinical monitoring visits in the SHC [32]. They will continue to fulfill this role during the study, augmenting their current work to manage HB-PrEP, and along with the lead research coordinator will help with eligibility screening, recruitment, and retention efforts.

### Primary RCT

#### Eligibility Criteria and Exclusions

All patients in the SHC meet both PrEP use criteria according to the US Centers for Disease Control and Prevention’s 2021 clinical practice guidelines [12] and at least one of three PHSKC PrEP criteria: (1) HIV-negative cisgender man, transgender or nonbinary person who has sex with men; (2) person who has a sex partner with unsuppressed HIV; or (3) person who injects drugs or engages in exchange sex [32]. Prospective participants for the trial, in addition, must meet the following study-specific criteria (Textbox 2).

Persons with active hepatitis B or a creatinine clearance <50 mL/minute are not excluded; however, they will receive counseling about the risk of hepatitis B infection worsening if PrEP medications are discontinued and be encouraged to follow up with medical providers outside of the study for management of comorbid conditions.

**Inclusion and exclusion criteria.**

**Inclusion criteria**

- Age ≥18 years.
- Resident of Washington State.
- Can speak, understand, read and write in English or Spanish.
- Current pre-exposure prophylaxis user or interested in starting or restarting pre-exposure prophylaxis.
- Willingness to be randomized to home-based pre-exposure prophylaxis and adhere to specified procedures.
- Willingness to provide primary and alternate contact information.

**Exclusion criteria**

- Recent (<4 weeks) “high risk” HIV exposure while off pre-exposure prophylaxis or symptoms of acute HIV infection.
- No mailing address to receive packages or sampling kits.
- No stable, working telephone number.
- No smartphone or electronic device with internet access.
- History of bleeding disorder, current or recent (≤7 days) use of anticoagulant medications (warfarin and rivaroxaban).
- Pregnancy.

#### Screening, Recruitment, and Enrollment

##### Existing Patients

DRIS or the research staff review patients scheduled for routine PrEP visits at the SHC and offer enrollment in person to eligible patients. Enrollees are randomized to continue SOC (attend the PHSKC SHC every 4 months for a visit with DRIS, have blood samples collected for PrEP monitoring, and perform self-collection of GC and CT swabs) or have the option to do HB-PrEP monitoring. Those assigned to HB-PrEP (intervention arm) are eligible to participate in home-based monitoring immediately after receiving instruction from study staff on how to use the Tasso devices, procedures for mailing kits back to the PHSKC laboratory, and how to complete web-based surveys after returning kits. DRIS or research staff facilitate training on the use of the Tasso devices. This includes a review of the test kit components, the importance of warming the upper arm, aseptic technique, and proper use of the device. After the overview, participants were also asked to watch the training video provided by Tasso (Tasso Inc) [30].

##### New and Former (“Lost to Follow-Up”) PHSKC PrEP Patients

Prospective and former PrEP patients who are eligible for the study may be recruited at their initial visit. Those randomized to the control arm will adhere to the usual SHC PrEP clinical protocols [32]. Persons randomized to HB-PrEP receive instruction from DRIS or study staff about self-collection procedures as above and are eligible to return their first kit for the month 4 monitoring cycle. Persons initiating PrEP (or reinitiating after a prolonged discontinuation) must be evaluated in person by a clinician and have blood collected by clinic staff for HIV screening, renal function, viral hepatitis serologies, and so on; thus, these individuals will become eligible to use home kits at their first tri-annual follow-up (ie, 120 days after PrEP initiation). Follow-up time in the study for all participants begins immediately after enrollment.

#### Randomization and Study Procedures

##### Sample Size and Power Considerations

Sample size calculations for the primary Kaplan-Meier survival analysis were based on standard methods [33]. Assuming that 2.5% of individuals discontinue PrEP per month in the control arm, we expect 45% of SOC participants to discontinue PrEP (55% retention) over the 20-month study period. In the intervention arm, we estimate that 70% will adopt HB-PrEP. We assume a relative risk of discontinuation of 0.6 among participants assigned to receive HB-PrEP leading to a 32% discontinuation rate (68% retention) in the HB-PrEP arm. We also allowed for an overall dropout rate in both groups of 6% [15]. Thus, a sample size of 458 participants should yield 80% power to detect a 13% point difference in PrEP retention. All eligible participants are assigned through simple 1:1 randomization to either the intervention arm (n=229) or SOC arm (n=229) and followed for 20 months. RCT procedures are detailed in Textbox 1.

##### Surveys

Participants in both arms are asked to complete web-based surveys using REDCap (Research Electronic Data Capture) [34]—a secure, web-based platform that participants used successfully in the preimplementation pilot study. The initial enrollment survey collects basic demographic and contact information including valid mailing address and phone number for the participant and an approved alternative contact, sexual orientation and gender identity items, and PrEP monitoring preferences. Subsequent interval surveys focus on evaluating self-reported PrEP adherence, number of sex partners, type and frequency of sex, frequency of drug or condom use, and measures of satisfaction with the assigned monitoring program.

##### Home Specimen Tracking

Participants in the HB-PrEP arm are contacted by study staff approximately 1 month before their visit (months 4, 8, and 16) to confirm whether they would prefer to come into the clinic or use a home sampling kit. Participants who opt for home sampling are sent a kit at that time to allow for samples to be received and results to be finalized before or near the date of their remote follow-up visit. Home sampling kits contain instruction sheets in English or Spanish, specimen labels, 2 swabs (Hologic Aptima 2 Combo, San Diego, California) for extragenital (or vaginal, if applicable) GC and CT testing, and 2 Tasso devices. The SHC’s protocol is not to offer men who have sex with men routine urine or urethral screening for GC and CT given that asymptomatic positivity at this anatomic site among men who have sex with men in our clinic is relatively rare and not felt to justify the cost of additional testing. Thus, consistent with our existing clinical protocols for in-person SOC, urine or urethral screening is not included in the home kits. Mailing and tracking of kits are managed through Tasso’s confidential kit delivery portal. Study staff enter participant information into the portal, at which point kits are assembled and mailed directly to the participant’s specified address. Tracking numbers allow staff to track the location of packages when they are received at the home, and when the participant arranges for pick-up to return specimens to the clinic. An automated text or email is then generated to notify participants that kits have been shipped and a reminder is sent 7 days after delivery if kits have not yet been returned.

##### Telehealth Visits and Interim Follow-Up

Participants in the HB-PrEP arm have 2 options for completing scheduled telehealth follow-ups: (1) through a telephone call with DRIS or (2) through a HIPAA (Health Insurance Portability and Accountability Act)-compliant Zoom conference call through the SHC electronic medical record. In addition, participants can reach study staff through WelTel—a secure and health information-compliant, 2-way message support program already in use by DRIS in our clinic for contacting PrEP patients [35]. Scheduled check-in visits for both arms consist of routine PrEP adherence counseling, screening for new HIV or STI symptoms, and PrEP prescription renewal if indicated. Per SHC protocols, participants with symptoms compatible with acute HIV or any STI must be evaluated in person in lieu of receiving a home sampling kit.

##### Specimen Processing and Laboratory Evaluations

Individuals randomized to HB-PrEP are mailed sampling kits to their preferred address and return self-collected samples through prepaid express postal mail to the PHSKC laboratory for processing within 48 hours. Participants are advised to refrigerate any sample that cannot be shipped and received in the clinic within 2 days (eg, over weekend days). Upon receipt in the clinic, the study staff and PI conduct a brief quality assessment of specimens, place laboratory orders in the electronic health record, and send samples to the PHSKC laboratory for testing per standard clinic protocol. Additional processing and testing procedures for HIV, syphilis, RPR, and creatinine have been described elsewhere [29].

#### Outcomes and Analyses

##### Primary Outcome Measures

Effectiveness will be evaluated with a survival analysis using PrEP nonretention as the event of interest. Observation time for all participants begins at the time of randomization and continues until either loss-to-follow-up or study end at 20 months. Consistent with previous studies [36,37], we define participants in either arm as “retained” on PrEP if they complete monitoring tests and visits with a clinician or DRIS at each 4-month interval to receive a renewed PrEP prescription. Kaplan-Meier curves will compare PrEP retention rates between the intervention and control arms at 20 months. We will use Cox proportional-hazards regression models to examine individual-level correlates (age, race and ethnicity, income range, and education status) of PrEP retention.

##### Secondary Outcome Measures

Reach is defined as the proportion of individuals assigned to the HB-PrEP study arm who choose to send in self-collected specimens from home for at least half of tri-annual PrEP visits over the study period. As we are interested in the real-world uptake of HB-PrEP, we are tracking and will report the total number of PrEP patients approached about the study, the number who declined participation and reasons for declination, and the proportion of individuals assigned to the intervention arm who choose at any time to transition to the SOC arm and reasons for doing so. To mitigate potential sampling (self-selection) bias, we explain in the screening process that even if randomized to the intervention arm, the use of HB-PrEP is optional, and patients in the intervention arm can always continue with standard in-clinic monitoring for some or all of the PrEP follow-up visits.

Acceptability or patient satisfaction with HB-PrEP will be evaluated using tri-annual web-based participant surveys including 5-point Likert scale questions adapted from questionnaires of similar PrEP trials. Additional items will inquire about the ease or difficulty of self-sampling device use, issues with receiving kits, and any other challenges with the home monitoring process. Specific validated psychometric tools to assess PrEP user satisfaction are limited overall and, to our knowledge, none exist that are designed to evaluate HB-PrEP services.

Study surveys inquire about PrEP use strategy (event-driven or “2-1-1” vs daily) over the preceding 4 months and adherence questions are tailored to the reported use strategy. Based on data that taking ≥4 doses/week confers a 96% reduction in HIV risk [38], self-reported PrEP adherence is measured using validated question items asking the percent of PrEP taken in the previous 4 weeks [39]. The percentage of PrEP adherence will be compared between arms and analyzed using linear mixed models to account for repeated measures on each participant.

We will measure the time from receipt of monitoring and STI results into the SHC electronic health record to the time study staff notify the participant of abnormal results.

We will also track new syphilis and GC or CT infections for each group per tri-annual interval to estimate a composite asymptomatic STI positivity incident rate.

##### Qualitative Data

Semistructured interviews will be conducted with HB-PrEP participants (n=24) beginning at month 4 and occur at triannual intervals thereafter, until recruitment goals are reached. The focus of these HB-PrEP participant interviews will be understanding specific barriers and facilitators of PrEP retention, identifying unanticipated negative effects or issues with the delivery of the intervention, determining whether HB-PrEP is preferred to SOC, and how future iterations of the program might be improved. Interview guides will be adjusted based on preliminary data from patient surveys to further explore any issues HB-PrEP participants identify. Clinic staff, including DRIS and administrators (n=3), will also be invited to participate in semistructured interviews at 12 and 20 months to understand operational barriers and facilitators of program implementation. Study participants and staff will be offered their choice of an in-person or virtual interview. Interviews will be audio recorded; transcripts will be independently reviewed by 2 study staff members and evaluated using thematic coding and a mix of inductive and deductive coding.

### Ethical Considerations

The study is considered human subjects research. All study procedures were reviewed and approved by the University of Washington institutional review board (IRB; STUDY00013871) and considered not to pose any more than minimal risk to study participants. Informed consent forms describing all study procedures, potential risks, compensation (if applicable), and contact information for the IRB and study staff were available in English and Spanish. Participation in the first study component (home-based monitoring vs SOC with web-based surveys) is not compensated. Patients and staff who participate in interviews for the qualitative study component will receive US $25 compensation.

All study staff are required to uphold strict confidentiality in accordance with the HIPAA. Only the information necessary to conduct the clinical trial or coordinate clinical care with providers will be viewed or used by authorized study staff. Home kits are in nonlabeled, plain boxes with no external information to identify study participants. Identifiable data is kept on an approved and password-locked database and only linked with indirect identifiers. Contributions in qualitative interviews will be deidentified and only general information used to derive themes for analysis.

## Results

### Overview

The HOT4PrEP trial is a hybrid implementation study that randomizes new or current HIV PrEP patients at the Sexual Health Clinic in Seattle, Washington, to have the option of home-based monitoring with self-collection of samples for HIV, syphilis, gonorrhea, chlamydia and creatinine and telehealth follow-up versus standard care including in-clinic visits and monitoring. The primary outcome is PrEP retention at 20 months.

Enrollment for the trial began in March 2022 and continued through May 2022 when all research was halted in the SHC to focus on the emerging mpox outbreak. Study enrollment then resumed in December 2022 and is ongoing.

From March 2022 to May 2023, a total of 161 PrEP patients were screened to enroll the first 100 participants into the intervention arm (49/100, 49%) or standard care arm (51/100, 51%). In total, 2 PrEP patients (1.2%) were deemed ineligible to enroll, 55 (34%) declined to participate, and staff were not able to discuss enrollment with 4 (2.5%) patients. Common reasons for declining to participate included preferring to come into the clinic or having an aversion to seeing blood during the self-collection process.

Table 1 describes demographics and other baseline characteristics participants self-reported upon enrollment. The baseline survey completion rate was 96% for both trial arms. Median age was 34 (IQR 28-39, range 19-67) years. Of the participants who elected to provide sex and gender information, all were assigned male sex at birth (47/47, 100%). Most identified as cisgender men (41/47, 87%), gay (42/47, 89%), and their race and ethnicity as non-Hispanic White (n=36, 38%) or Asian (n=23, 24%). Most participants reported an annual income of US $30,000 or more (75%) and at least some college education (91%).

All participants in the intervention arm were contacted before their 4-month follow-up visit to ask if they would prefer to come into clinic or receive home kits. Of the 49 participants contacted, 33 (67%) opted to collect samples at home and follow up through telehealth, 14 (29%) opted to come in person to the clinic, and 2 (4%) canceled their appointments. Of those who opted for home sample collection and telehealth follow up, 27 (82%) returned test kits with samples for HIV and STI testing, 5 (15%) came in person to the clinic for their visit, and 1 (3%) canceled their appointment.

**Table 1:** Baseline characteristics of the first 100 home option testing for pre-exposure prophylaxis trial enrollees, March 2022-May 2023.

Characteristics		Values
Age (years), median (IQR)		34 (28-39)
Male sex assigned at birth, n (%)		47 (47)
**Current gender identity, n (%)**		
	Cisgender man	41 (87)
	Transgender, nonbinary, genderqueer, etc	6 (11)
**Race and ethnicity, n (%)**		
	NH^a^ Black	10 (11)
	Hispanic or Latinx	15 (16)
	NH^a^ White	36 (38)
	Asian	23 (24)
	American Indian or Alaska native, mixed race, or other	8 (9)
**Sexual orientation, n (%)**		
	Gay	42 (89)
	Straight, pansexual, bisexual, or queer	5 (11)
**Annual income (US$), n (%)**		
	14,999 or less	5 (11)
	15,000-29,999	6 (14)
	30,000-49,999	11 (25)
	50,000-99,999	11 (25)
	100,000 or more	11 (25)
**Highest level of education, n (%)**		
	Grade or high school	4 (8)
	Some college, associate, or technical degree	11 (23)
	Bachelor’s degree	17 (35)
	Some graduate school	5 (11)
	Graduate degree	11 (23)
**Medical insurance (excluding PrEP^b^ care), n (%)**		
	None	17 (37)
	Private insurance	24 (52)
	Medicaid	2 (4)
	Other insurance type	3 (7)
**Ever diagnosed with STI^c^, n (%)**		
	Yes	33 (70)
	No	14 (30)
**PrEP^b^ use status, n (%)**		
	Currently taking PrEP^b^	42 (89)
	Planning to start soon	5 (11)
**Duration of current PrEP^b^ use, n (%)**		
	<3 months	3 (7)
	3-6 months	8 (20)
	6-12 months	6 (14)
	1-2 years	8 (20)
	>2 years	16 (39)
**Substance use in the past 12 months^d^, n (%)**		
	Alcohol	37 (37)
	Stimulants (cocaine or meth)	9 (9)
	Hallucinogens (LSD or mushrooms)	6 (6)
	Marijuana	25 (25)
	None	5 (5)

^a^NH: non-Hispanic.

^b^PrEP: pre-exposure prophylaxis.

^c^STI: sexually transmitted infections.

^d^Total percentages may not sum to 100% as participants could select multiple answer choices.

## Discussion

### Principal Findings

Preliminary data from the first 100 participants of this hybrid RCT suggest that thus far, the implementation strategy of HB-PrEP monitoring, consisting of self-collected GC and CT swabs, blood specimens obtained using the Tasso device, and telehealth follow-up, is acceptable to users and feasible to incorporate into a busy PrEP clinic. Though results are preliminary, reach appears to be high among participants randomized to the intervention arm. Two-thirds of participants who were offered the option of HB-PrEP chose to use it, and of those who received kits, 27 (82%) successfully returned kits containing samples sufficient to complete their requisite PrEP monitoring laboratory tests.

Our initial pilot study undertaken during the COVID-19 pandemic (2020-2021) found that 98 (80%) of surveyed patients in our clinic preferred home-based services over coming into the clinic. Some studies conducted in the prepandemic era [22,23,40] and another completed more recently among men who have sex with men in a Dutch sexual health center [41] came to similar conclusions about the preference for home-based sampling. However, at least 1 other study conducted in a real-world pharmacy-based PrEP program found that less than half of participants used home kits when offered them [26]. Factors affecting a participants’ decision to do home sampling over coming to the clinic may be variable and depend on one’s self-efficacy and assessments of potential risks (eg, breach of privacy) versus benefits (eg, increased convenience and saved time) [26]. While our preliminary results of home kit uptake are promising, it remains to be seen if stated preference or intention to self-collect samples at home will equate to consistent use over time. Survey data and interviews of participants will aim to identify reasons motivating choice over time.

Our trial faces a few challenges and limitations. Interpretation of preliminary results remains limited given that enrollment in the randomized portion of the trial is ongoing. Study staff are recruiting for the qualitative component of the study, and analyses of preliminary PrEP retention rates at 4 and 8 months and secondary outcomes are also underway. While the initial cohort is diverse from a racial and ethnic standpoint, other demographics and characteristics may not represent groups of PrEP patients in other settings. Our clinic is large, serves an urban population, and is fairly well-resourced; thus, results may not be generalizable to smaller clinics in less populous or less well-resourced areas. We will not be able to verify that returned samples came from the enrolled participant, but we do not anticipate this to be a significant problem since abnormal or positive test results will require an in-person clinic visit for confirmatory retesting and treatment. Finally, the first component of our RCT is not incentivized which may be associated with decreasing engagement over time with study procedures (eg, survey completion). However, this trial was designed to be pragmatic with study procedures that could be reproducible for similar clinics where routine PrEP care is not incentivized in the real world. Despite these challenges, we feel the study seeks to answer important questions and will yield useful information about the utility, barriers, and facilitators of this implementation strategy for expanding PrEP access locally and beyond.

While not every person may choose to use a home-based option for every PrEP follow-up visit, HB-PrEP holds promise as a strategy to decrease barriers for staying on PrEP, increase patient autonomy and accessibility, and expand the capacity of clinics to initiate and maintain a greater number of people on PrEP. In turn, if successful even for a proportion of users, HB-PrEP has the potential to contribute to larger efforts to end the HIV epidemic by increasing PrEP uptake and retention. Our results will provide important information about the feasibility and value of implementing HB-PrEP into growing programs seeking to strike a balance between expanding PrEP access, maintaining capacity for in-person services, and facilitating patient choice and convenience.

## Abbreviations

CT: chlamydia
DRIS: disease and research intervention specialist
GC: gonorrhea
HB-PrEP: home-based pre-exposure prophylaxis
HIPAA: Health Insurance Portability and Accountability Act
HOT4PrEP: home option testing for pre-exposure prophylaxis
IRB: institutional review board
PHSKC: Public Health-Seattle and King County
PrEP: pre-exposure prophylaxis
RCT: randomized controlled trial
REDCap: Research Electronic Data Capture
RPR: rapid plasma reagin
SOC: standard of care
SHC: sexual health clinic
STI: sexually transmitted infection

## Data Availability

The data sets generated during and analyzed during this study are not publicly available as study enrollment is ongoing and requires the collection of data involving protected health information about notifiable conditions. Unidentified data are available from the corresponding author on reasonable request.
